# Nonlinear age effects on basketball player performance: insights from Kolmogorov–Arnold Networks in NBA data

**DOI:** 10.3389/fspor.2025.1693433

**Published:** 2025-11-03

**Authors:** Yunhan Xiao, Jiahao Wang, Weiping Li, Jiangang Chen, Ning Chang, Yilong Song, Ziying Xu

**Affiliations:** ^1^ Department of Sports and Health Science, Xi'an Physical Education University, Xi'an, Shaanxi, China; ^2^ School of Physical Education and Training, Xi'an Sports University, Xi'an, Shaanxi, China

**Keywords:** Kolmogorov–Arnold Networks, basketball performance prediction, machine learning, nonlinear modeling, age-related performance

## Abstract

**Introduction:**

This study utilizes 2,786 NBA player–season samples from 2019 to 2024 to develop a nonlinear modeling approach based on Kolmogorov–Arnold Networks (KAN), applied to modeling the relationship between player age and basketball performance. A novel modeling framework is proposed, integrating interpretable machine learning with age-group-specific feature analysis, aiming to systematically reveal the nonlinear dynamics and transitional mechanisms of performance evolution across age.

**Methods:**

Fantasy Points is used as the unified performance metric, and players are categorized into three age groups: Youth (19–23 years), Prime (24–30 years), and Veteran (31–40 years). The KAN model is tuned via Bayesian optimization and evaluated using five-fold cross-validation. Its performance is systematically compared against mainstream models, including Multilayer Perceptron (MLP), XGBoost, Random Forest, and Linear Regression.

**Results:**

Results show that KAN achieves the lowest MAE and RMSE across all age groups, with the best or near-best R² values. In the youth group, the model achieves MAE = 0.089, RMSE = 0.115, and R² = 0.986, significantly outperforming all baseline models. Further response function analysis reveals nonlinear structural features in the age–performance relationship. Attribution results indicate that youth performance is driven by multiple interacting variables with strong and volatile marginal effects; in Prime, performance stabilizes and is dominated by key metrics such as points (PTS), assists (AST), and rebounds (REB); in Veteran, performance converges on a few core variables, with a “ceiling effect” and diminishing marginal returns.

**Discussion/Conclusion:**

Using a KAN-based nonlinear framework, we reveal the age-group-specific evolution of basketball performance with age, offering new methodological insights for career management, training optimization, and intelligent decision-making in professional sports.

## 1 Introduction

Long-term athlete development pathways are a key topic in sports science and training management. The “Long-Term Athlete Development” (LTAD) framework indicates that athletes must undergo systematic training and skill accumulation starting from adolescence, gradually reaching their competitive peak, followed by a decline in abilities with advancing age, forming a multi-stage, nonlinear developmental trajectory (1–4). In most sports, peak performance age typically falls between 20 and 30 years old (5–7). Taking basketball as an example, players usually reach their competitive peak in their late 20s (around 27 years old), with performance beginning to decline after 30 (8). However, the relationship between peak performance and decline is not simply linear. Athletes' performance at different age stages is influenced by multiple factors such as physiology, technique, and experience, exhibiting complex nonlinear changes. As age increases, physical attributes like explosiveness and speed gradually decline, but technical skills, tactical understanding, and experience may improve, allowing some players to maintain high efficiency beyond 30 (8, 9). Nonlinear modeling results also show that athletes' physical and technical changes across age groups follow multidimensional, asynchronous trajectories, which single linear models struggle to explain (3, 10). Therefore, understanding the multi-stage, nonlinear characteristics of athlete performance with age is crucial for scientifically planning training and optimizing career management.

The relationship between age effects and athletic performance is complex and variable, difficult to describe with simple linear relationships. It is both a dynamic multi-stage process and the result of multifactorial interactions, influenced by physiological, technical, psychological, and experiential factors (6). Due to this complexity, previous research has often approached the topic from the perspective of the Relative Age Effect (RAE), exploring the heterogeneity and mechanisms between age and athletic outcomes. For instance, Musch et al. systematically reviewed the prevalence and mechanisms of RAE across various sports, noting that minor differences in birth months can significantly impact selection, development opportunities, and competitive levels (11). Wattie et al. proposed a developmental systems model, further revealing the multifaceted factors behind RAE (12). Specific to basketball, Ibáñez et al. found that RAE differentially affects performance based on playing positions in U18 athletes (13). However, traditional RAE analyses rely primarily on manual statistics and grouping methods, limiting evaluation efficiency. Thus, there is a need for data-driven, automated dynamic analysis tools based on big data to enhance assessment accuracy and efficiency. Analyses of athlete performance data and technical features have already enabled effective identification of different developmental stages and competitive levels (14, 15). Therefore, employing machine learning methods for dynamic modeling and prediction of these features offers new pathways for continuous monitoring and management of athletic performance.

Kolmogorov–Arnold Networks (KAN), as an emerging neural network model based on the Kolmogorov–Arnold representation theorem, was proposed by Liu et al. (16). Unlike traditional Multi-Layer Perceptrons (MLPs), KAN incorporates learnable edge activation functions (such as B-splines) in its network structure, providing stronger nonlinear approximation capabilities and higher interpretability. Studies show that KAN outperforms MLPs and ensemble tree models (e.g., XGBoost) in tasks like function regression, solving differential equations, and physical field modeling, offering structural explanations of the prediction process (17). Similarly, KAN has been successfully applied in function regression and physical field modeling in complex nonlinear systems, demonstrating its broad potential in multivariable dynamic prediction problems (18, 19). However, KAN has not yet been applied in sports, particularly in basketball performance modeling and analysis. Given KAN's excellence in nonlinear modeling of complex systems, introducing it to dynamic analysis of athlete performance data could achieve high-precision prediction and interpretable modeling of performance trajectories, providing new theoretical and technical support for basketball athlete training and development.

Despite prior explorations of the relationship between age and athletic performance, most studies are limited to linear regression, manual segmentation, or traditional statistical analysis, falling short in revealing the nonlinear and multi-stage features of performance evolution. In team sports like basketball, research often focuses on adolescents, lacking systematic examination of performance change patterns and influencing factors across all age stages, resulting in limited understanding of athletes' full lifecycle development. This highlights the need for a method that dynamically captures nonlinear changes and systematically analyzes performance patterns and key factors at different ages, to more comprehensively understand athletes' lifelong development processes.

This study's innovation lies in being the first to introduce Kolmogorov–Arnold Networks (KAN) to dynamic modeling of basketball athlete performance. Leveraging KAN's powerful nonlinear fitting and high interpretability, we systematically analyze the trajectory of age's impact on performance. Unlike previous reliance on linear or traditional statistical models, this paper uses NBA Fantasy Points as a unified and comprehensive performance metric, based on large-scale real data, comparing KAN with mainstream machine learning models (e.g., MLP, XGBoost). By doing so, we aim to more accurately capture the nonlinear features and multi-stage trajectories of athlete performance with age, revealing patterns and key influencing factors at different stages, thereby providing scientific evidence and methodological innovation for athlete lifecycle management and personalized training program development. The importance of this study is multifaceted: theoretically, it advances the LTAD framework by quantifying nonlinear performance mechanisms; practically, it offers data-driven insights for coaches in age-specific training, career planning, and management; methodologically, it validates KAN's prospects in sports big data, potentially extending to injury prediction or team strategy optimization. According to existing literature and tool search results (such as arXiv), KAN's application in sports performance analysis remains in an emerging stage (20, 21), and this paper may be the first systematic empirical study applying KAN in basketball, further highlighting its innovation and significance.

To enable comprehensive quantification and cross-comparison of basketball athletes' competitive performance, this paper selects NBA official Fantasy Points as a unified performance indicator. This metric weights and integrates key data such as points, rebounds, assists, steals, and blocks into a single value, objectively and holistically reflecting players' overall contributions to games. Compared to methods evaluating players based on single stats (e.g., points, efficiency), Fantasy Points provide a more comprehensive, fair assessment of players across positions and types, avoiding bias and enhancing data comparability (22, 23).

This paper proposes a KAN-based dynamic modeling method for basketball athlete performance, aiming to systematically reveal the nonlinear influence patterns of age on athletic performance. The main contributions of this study include: 1. Proposing a KAN-based dynamic modeling method for basketball athlete performance: Introducing Kolmogorov–Arnold Networks (KAN) to the basketball domain for the first time to systematically model and analyze nonlinear patterns of performance with age, enhancing modeling capabilities and interpretability for complex dynamic processes. 2. Innovative application of a comprehensive performance metric: Using NBA Fantasy Points as a unified indicator enables cross-comparable, multidimensional evaluation of players across positions and types, providing new measurement tools for athlete performance research. 3. age-group-specific analysis and multi-model comparison: Based on theoretical and empirical foundations, grouping players by age groups reveals heterogeneity in performance changes; comparisons with MLP, XGBoost, etc., validate KAN's advantages in dynamic modeling and key feature identification. The remainder of this paper is organized as follows: Section 2 describes data sources, performance indicators, and sample grouping methods; Section 3 details the KAN and comparative models' building processes and experimental design; Section 4 reports empirical results and analysis; Section 5 summarizes contributions, limitations, and future research prospects.

This study, by introducing the Kolmogorov–Arnold Networks (KAN) model, advances the optimization of nonlinear modeling and analysis of basketball athlete competitive performance. This method holds potential application prospects in athlete development assessment, personalized training optimization, and intelligent performance analysis in sports.

## 2 Data sources and acquisition

In this study, player performance data from the 2019–2020 to 2023–2024 NBA regular seasons were collected using automated Python scripts built with the nba_api library (https://github.com/swar/nba_api), which interfaces with the official NBA data source. All available player statistics across five complete seasons were retrieved. To ensure data reliability and reproducibility, the script was programmed to iteratively request data for each season with appropriate time delays to prevent data loss due to excessive request frequency. Each entry was also automatically labeled with the corresponding season identifier.

Following initial acquisition, the dataset underwent standardized cleaning procedures. Invalid players, extreme outliers, and records with missing or incomplete information were removed. The final structured dataset includes 2,786 player-season samples, comprising basic personal information, season-level technical statistics, and composite performance metrics.

This dataset does not involve any human or animal subjects, nor does it contain any personally identifiable or sensitive information. Therefore, no ethical review or institutional approval was required.

### 2.1 Age grouping and variable definitions

Based on established theories and empirical research in sports science, NBA players in this study were categorized into three age groups:(1) Youth (19–23 years), (2) Prime (24–30 years), and (3) Veteran (31–40 years).

This classification draws on the Long-Term Athlete Development (LTAD) model proposed by Balyi and Way, as well as the systematic review of peak athletic performance age conducted by Allen et al., both of which suggest that most athletes reach peak performance between the ages of 20 and 30, followed by a gradual decline thereafter (1, 6). This segmentation approach has been widely validated in the existing sports science literature and is applicable to basketball as well as many other sports (3, 24).

The prediction task focuses on modeling NBA players' multi-season composite performance, operationalized using NBA Fantasy Points, which constitutes a continuous regression problem. All input and output variables were constructed based on a thorough understanding of basketball performance dynamics and athlete evaluation characteristics, while also accounting for data structure and the practical significance of statistical indicators.

Specifically, the output variable is NBA_FANTASY_PTS, representing a player's season-level composite performance. This metric integrates multiple key performance statistics—such as points, rebounds, assists, steals, blocks, and turnovers—into a single score, calculated according to standard fantasy scoring systems. It offers an effective measure of a player's overall contribution. The calculation formula is presented in Equation (1).

(1) NBAFantasyPoints=PTS+1.2×REB+1.5×AST+3×STL+3×BLK−1×TOV

We use a publicly available Fantasy scoring scheme as the target metric, composed of weighted countable events with positive weights for key offensive and defensive actions and a negative weight for turnovers; it provides a convenient unified summary of performance but is not position-neutral and only partially covers off-ball and coordination contributions. Accordingly, the age-related nonlinear patterns discussed below should be interpreted as relative effects under this weighting lens rather than as a complete characterization of overall player value.

The input variables primarily include players' season-level fundamental technical statistics, such as:points per game (PTS), field goal percentage (FG_PCT), three-point field goals made (FG3M), assists (AST), rebounds (REB), steals (STL), blocks (BLK), and turnovers (TOV).

Considering potential differences in scale and distribution among these variables, all input features were standardized prior to model training to improve stability and enhance the generalization performance of the models.The definitions of these variables are provided in Supplementary Table S1.

### 2.2 Model construction and analytical procedure

#### 2.1.1 Data preprocessing

To enhance model generalization and interpretability, this study conducted systematic data preprocessing prior to model training. First, all raw feature variables were examined for missing values and outliers, which were appropriately handled to ensure data integrity. Subsequently, to eliminate extreme or unrepresentative samples, we excluded player-season records in which the player averaged fewer than 10 min or fewer than 10 points per game, thereby improving the robustness and representativeness of the dataset.

This exclusion criterion was intended to reduce the influence of fringe players, those recovering from injuries, or short-term signees—players whose playing time is often inconsistent and whose performance tends to fluctuate considerably. Such records typically exhibit high statistical randomness, which may introduce noise and hinder the convergence and generalization of predictive models. Similar sample filtering strategies have been employed in previous studies. For instance, research using data mining techniques to analyze NBA player performance excluded players with limited minutes to avoid overfitting to outlier behaviors (24). Other studies using Bayesian modeling to investigate age-related performance trajectories also emphasized the need to exclude statistically unstable individual samples in order to improve the accuracy of nonlinear curve estimation (25).

To better understand the relationships among technical indicators and to optimize input feature selection, we performed an exploratory correlation analysis of all relevant variables. Pearson correlation coefficients were calculated to assess pairwise linear relationships, and results were visualized using a correlation heatmap. In the heatmap, color intensity reflects the strength of correlation: darker shades indicate stronger positive correlations, while lighter shades represent stronger negative correlations. Each cell is labeled with the corresponding correlation coefficient between two features. An example heatmap based on full-game technical statistics is shown in Supplementary Figure S1.

The analysis revealed high correlations among several feature pairs (e.g., FGM and PTS: r = 0.99, FG3M and FG3A: r = 0.98, MIN and PTS: r = 0.88, AST and TOV: r = 0.84). To mitigate feature redundancy and multicollinearity, we applied a correlation threshold of |r| ≥ 0.8. For each highly correlated feature pair, we retained the more representative variable—one that independently reflects player ability—such as FGM, FG3M, and FTM. Highly correlated features such as PTS, FGA, and FG3A were removed accordingly (see Supplementary Table S2 for details).

However, given the strategic and statistical importance of scoring, offensive rebounds, and defensive rebounds in determining both individual and team performance—and their well-established association with game outcomes and athletic effectiveness (26, 27)—we chose to retain all 15 core technical indicators in the final model, including both offensive and defensive rebounds.

#### 2.2.2 Construction of the KAN model

Kolmogorov–Arnold Networks KAN approximate multivariate relationships by learning one-dimensional smooth functions on edges and additively composing them to form the output. Unlike MLPs that stack fixed activations at nodes, KAN places learnable activations on edges, achieves strong nonlinearity with shallow depth, and produces interpretable univariate response curves for each predictor that reveal thresholds, plateaus, and diminishing returns. Within a five-fold cross-validation framework, we used Bayesian optimization to search hyperparameters including depth, width, learning rate, and weight decay, and we applied early stopping that terminates training when the validation set shows no improvement for several consecutive epochs.

To examine the nonlinear influence of age on basketball player performance, we adopt KAN for predictive modeling and analysis. Supplementary Figure S2 presents the model architecture. To maximize predictive performance, we tuned KAN and several mainstream baseline models—Multilayer Perceptron MLP, XGBoost, Random Forest RF, and Support Vector Machine SVM—using Bayesian optimization and grid search. Under a five-fold cross-validation framework, models were compared using Mean Absolute Error MAE, Root Mean Square Error RMSE, and Coefficient of Determination R^2^. Results show that KAN outperformed the baselines in capturing complex nonlinear relationships while providing superior interpretability.

The structure of KAN is grounded in the Kolmogorov–Arnold representation theorem, which states that any continuous multivariate function can be expressed as a finite sum of continuous univariate functions. Accordingly, KAN parameterizes learnable univariate activation functions on edges and forms the overall output through additive mixing. The matrix form used in this study is given in Equation 2.

(2) $$f(x)=\Phi_{\mathrm{out}}\circ \Phi_{\mathrm{in}}\circ x$$

Here, $\Phi_{\mathrm{in}}$ and $\Phi_{\mathrm{out}}$ represent the function matrices of the input and output layers, respectively, as shown in Equation (3).

(3) $$\Phi_{\mathrm{in}}=\left(\begin{array}{ccc} \phi_{1,1}(\cdot ) & \cdots & \phi_{1,n_{\mathrm{in}}}(\cdot )\\ \vdots & \ddots & \vdots \\ \phi_{n_{\mathrm{out}},1}(\cdot ) & \cdots & \phi_{n_{\mathrm{out}},n_{\mathrm{in}}}(\cdot ) \end{array}\right),\;\Phi_{\mathrm{out}}=(\Phi_{1}(\cdot )\cdots \Phi_{n_{\mathrm{out}}}(\cdot ))$$

In the practical implementation of KAN, the numbers of input and output nodes are typically set as $n_{\mathrm{in}}=n$, $n_{\mathrm{out}}=2n+1$, respectively. After multiple nested layers, the overall mapping function of a KAN network with LLL layers can be expressed as shown in Equation (4).

(4) $$\mathrm{KAN}(x)=\Phi_{L-1}\circ \cdots \circ \Phi_{1}\circ \Phi_{0}\circ x$$

The training objective of the KAN model is to minimize the error between the predicted and actual values, while incorporating a regularization term to control model complexity and prevent overfitting. The loss function is defined as follows:

$$L=\frac{1}{N}\overset{N}{\underset{i=1}{\sum }}\;y_{i}-{\hat{y}}_{i}^{2}+\lambda \Omega (\Phi )$$

Where $y_{i}\;$ is *i* the true label of the *i*th sample, ${\hat{y}}_{i}\;$ is the predicted output of the KAN model, *N* is the total number of samples, λ is the regularization coefficient, and Ω(Φ) is the penalty term for the smoothness of univariate functions in the network (such as B-splines) (e.g., the square of the second derivative).

To ensure model reproducibility and avoid potential data leakage, this study implemented the KAN model using the pykan library. The specific architecture settings are as follows: width = [15, hidden_size, 1] (input layer with 15 features, hidden layer with hidden_size nodes, output layer with 1; hidden_size ranges from 8 to 32, obtained from optimization), depth (ranging from 2 to 6, obtained from optimization), grid size grid = 5 (default), spline order *k* = 3 (default), random seed seed = 0 (for optimization random state), regularization coefficient weight_decay (ranging from 1e-6 to 1e-2, log-uniform distribution, obtained from optimization). The optimizer is Adam, with learning rate lr (ranging from 1e-4 to 1e-2, log-uniform distribution, obtained from optimization). Training for up to 200 epochs, using an early stopping mechanism (patience = 15, minimum improvement threshold for validation loss 1e-5). The loss function is MSELoss. Hyperparameter optimization uses scikit-optimize's gp_minimize function, with n_calls = 15, random_state = 0. Under each hyperparameter combination, 5-fold CV evaluates the average MAE as the objective function. Within each fold, randomly split 90% sub-training set and 10% validation set from the training set (no fixed seed, but overall KFold has random_state = 42) for early stopping.

In the five-fold cross-validation, data splitting is performed using sklearn.model_selection.KFold(n_splits = 5, shuffle = True, random_state = 42) to ensure randomness and reproducibility. Feature standardization is performed independently in each fold (using sklearn.StandardScaler, fit only on training data, then transform validation/test data) to prevent information leakage from the training set to the validation/test set. At the same time, hyperparameter tuning is performed only within the training folds to avoid cross-fold leakage. Finally, the optimal hyperparameters are used for the complete 5-fold CV evaluation.

## 3 Results

### 3.1 Model performance comparison

To comprehensively evaluate the effectiveness of the KAN model in predicting season-level performance of basketball players, we conducted a systematic comparative analysis with several mainstream machine learning models, including Multilayer Perceptron (MLP), Extreme Gradient Boosting (XGBoost), Random Forest (RF), and Support Vector Machine (SVM) as baseline models. To ensure fairness in comparison, all experiments were performed using the same dataset, identical feature selection, and standardized preprocessing procedures. Bayesian optimization was applied for automated hyperparameter tuning, and five-fold cross-validation was uniformly employed across all models to ensure the robustness and comparability of results.

During the five-fold cross-validation, we used three key regression metrics to assess model performance: Mean Absolute Error (MAE), Root Mean Squared Error (RMSE), and Coefficient of Determination (R^2^).MAE reflects the average absolute deviation between the predicted and actual values. A smaller MAE indicates more accurate overall predictions. RMSE measures the square root of the mean of squared prediction errors and is more sensitive to large deviations; a lower RMSE indicates better model fit.R^2^ evaluates how well the model explains the variance in the target variable. An R^2^ value closer to 1 implies greater explanatory power and better goodness of fit.

$$\begin{array}{c} \mathrm{MAE}=\frac{1}{N}\overset{N}{\underset{i=1}{\sum }}\;\;|y_{i}-{\hat{y}}_{i}| \end{array}$$

$$\begin{array}{c} \mathrm{RMSE}=\sqrt{\frac{1}{N}\overset{N}{\underset{i=1}{\sum }}\;\;{\left(y_{i}-{\hat{y}}_{i}\right)}^{2}} \end{array}$$

$$\begin{array}{c} R^{2}=1-\frac{\sum_{i=1}^{N}\;\;{\left(y_{i}-{\hat{y}}_{i}\right)}^{2}}{\sum_{i=1}^{N}\;\;{\left(y_{i}-\bar{y}\right)}^{2}} \end{array}$$

Where $y_{i}$ denotes the ground truth value, ${\hat{y}}_{i}$ is the predicted value by the model, $\bar{y}$ is the mean of the ground truth values, and *N* is the total number of samples.

#### 3.1.1 Five-fold cross-validation comparative experiment

After systematic hyperparameter tuning using Bayesian optimization, the predictive performance of KAN, MLP, XGBoost, Random Forest, and Linear Regression models across different age groups is presented in Supplementary Table S3–S5 and visualized in Supplementary Figure S5. The results show that across all age categories, the KAN model consistently achieved the lowest values in both Mean Absolute Error (MAE) and Root Mean Squared Error (RMSE), while also reaching or closely approaching the best performance in Coefficient of Determination (R^2^)—demonstrating a clear and consistent advantage.

For example, in the 19–23 age group, the KAN model achieved MAE = 0.0890, RMSE = 0.1152, and *R*^2^ = 0.9855, significantly outperforming all other baseline algorithms.

Notably, in the Veteran (31–40 years) group—where sample size is smaller and performance variation is higher—the KAN model still maintained stable predictive superiority, indicating strong generalization capability. Overall, KAN effectively models the complex nonlinear relationships between players' technical statistics and overall performance, and outperforms traditional neural networks and ensemble tree models in comprehensive five-fold cross-validation assessments.

### 3.2 Analysis of key performance drivers across age groups

To further investigate the age-related heterogeneity in basketball player performance, we applied feature attribution techniques based on the KAN model to identify the most influential technical statistics for each age group. The results are presented in Supplementary Figure S4.

Supplementary Figures S4d–f display the feature attribution distributions derived from the KAN model for players in the 19–23, 24–30, and 31–40 age groups, respectively. Overall, the results reveal a dynamic evolution of key performance drivers as players age.

In the 19–23 age group (Supplementary Figure S4d), offensive metrics such as assists (AST), points scored (PTS), and field goal attempts (FGA) received the highest attribution weights, indicating that younger players rely more heavily on offensive production to drive overall performance.In the 24–30 age group (Supplementary Figure S4e), the attribution weight of PTS increased significantly, making it the most critical factor influencing season-level performance. AST and rebounds (REB) followed closely, suggesting that players in this age group contribute both as primary scorers and all-around performers.In the 31–40 age group (Supplementary Figure S4f), PTS, AST, and REB remained the top contributors, but the overall attribution became more evenly distributed. Interestingly, the relative importance of defensive indicators such as blocks (BLK) and steals (STL) increased slightly—indicating that Veteran players, beyond relying on scoring, tend to maintain their impact through experience-based defensive contributions.

#### 3.2.1 Visualization of nonlinear attribution structures across age groups

To further characterize the nonlinear mechanisms underlying player performance across different age groups, we visualized both the pruned structures of the KAN networks and the univariate response functions of dominant input features for players in the 19–23, 24–30, and 31–40 age groups.

From the pruned KAN structures, we observed that the network for players aged 19–23 retained a relatively large number of active branches even after pruning. This indicates that player performance at this stage is driven by a diverse set of technical abilities, with a non-converged skill structure—reflecting high developmental potential and flexibility. In contrast, the 24–30 age group exhibited a more simplified KAN structure with fewer dominant features, suggesting that technical abilities become more stabilized and performance is increasingly shaped by a smaller set of key variables. For the 31–40 age group, the structure further converged toward a minimal number of dominant features.

In the corresponding univariate response function plots (shown in the smaller panels to the right), we found:19–23 Age Group (Supplementary Figure S5): Dominant features such as PTS, AST, FGA, REB, FG_PCT, and STL exhibit strong nonlinear relationships. For example, the response function for PTS shows an increasing marginal effect, indicating that gains in scoring have a disproportionately large positive impact on overall performance. Functions for AST and REB display inflection points or fluctuations, suggesting threshold effects or synergy-based dynamics. Overall, younger players show high sensitivity to multidimensional skill development, with substantial room for growth.

24–30 Age Group (Supplementary Figure S6): The response functions for key variables—such as PTS, AST, and FG_PCT—tend to become linear or near-linear, with smaller slopes. This implies diminishing marginal returns: further improvements in these core skills yield smaller gains in overall performance. Player output during this stage stabilizes, relying more on solidified skillsets and accumulated experience, with less interaction among technical variables.

31–40 Age Group (Supplementary Figure S7): Dominant features further converge to a very small set (e.g., PTS, FG_PCT, AST), whose response functions mostly exhibit plateaus or slight declines. In many cases, marginal effects vanish entirely. This indicates a clear “ceiling effect”, where most players have reached their limits in key abilities, and improvements in overall performance depend almost exclusively on a few stable technical attributes.

These KAN visualizations reveal the age-specific nonlinear response patterns between technical features and overall player performance: Youth (19–23 years) display multidimensional, highly sensitive performance responses with significant development potential; Prime (24–30 years) show performance stabilization with reliance on core skill maintenance; Veteran players exhibit high dependence on a limited number of stable technical attributes, with evident convergence in performance-driving features.

## 4 Discussion

This study employs Kolmogorov–Arnold Networks, KAN, to explore the nonlinear characteristics and key performance drivers of NBA players across three age groups: youth 19–23, prime 24–30, and veteran 31–40. The results show that KAN significantly outperforms traditional machine learning models such as MLP, XGBoost, Random Forest, and Linear Regression, in both predictive accuracy and interpretability. This finding is consistent with recent work by Liu and Vaca-Rubio et al., who highlight KAN's strengths in modeling complex nonlinear systems (16, 17). More importantly, beyond prediction, we convert KAN outputs into guidance that coaches and players can execute directly: treat attribution weights as each player's “primary feature list,” and use the slope, inflection point, and plateau of the univariate response functions as signals for training and deployment decisions. In practice, the coaching staff can act on the KAN curves as follows: when a primary feature such as PTS, AST, REB, or FG_PCT exhibits a steep positive local slope, moderately increase training and in-game usage related to that feature; when the current level approaches an inflection region, adopt short, threshold-oriented training blocks, such as pairing high-intensity shooting with decision-making simulations, to help cross typical thresholds or bottlenecks, such as AST 3–5 and REB 4–6; when the curve enters a plateau or marginal effects weaken, shift to efficiency maintenance and load management, such as shooting-percentage maintenance and defensive positioning, to avoid inefficient expansion.

Our analysis reveals three age groups: Youth (19–23 years): Performance in this stage is strongly influenced by multiple key technical indicators—including PTS, AST, REB, and STL—with pronounced nonlinear marginal effects. This aligns with prior research showing that Youth (19–23 years)’ technical and physical development is multidimensional, dynamic, and nonlinear (28, 29). For example, Höhn et al. (2022) used big data and Bayesian analysis to show that young NBA players' growth is driven by the synergy of multiple technical abilities and exhibits clear nonlinear trajectories (30). For coaches: when a youth player's PTS/AST/REB response curves show a clear positive slope at the current level, increase training and usage tied to those features to amplify marginal gains; when AST or REB approaches the indicated threshold ranges (about 3–5 assists or 4–6 rebounds per game), implement short training cycles and evaluate against whether the threshold is reached, observing whether an accelerated segment emerges.

Prime (24–30 years): Technical attributes become markedly more stable and linear. Response functions for primary features (e.g., PTS, AST, REB, FG_PCT) are closer to linear, and marginal contributions tend to stabilize. This reflects the establishment of efficient skill systems in which overall performance relies more on coordinated and consistent output of core skills. Studies by Kalén et al. confirm that performance in this stage is more predictable and enters a “technical stability plateau,” with reduced volatility (8, 25, 29). Accordingly, when the KAN curves are near-linear with small slopes above a high baseline (e.g., >15 points or >5 assists per game), training and deployment should shift from expansion to efficiency maintenance and coordinated optimization: use FG_PCT and AST/TO as efficiency anchors, maintain coordinated output in PTS/AST/REB, and avoid additional investment in single items with diminishing returns.

Veteran (31–40 years): Players exhibit a clear “ceiling effect,” and the KAN network structure becomes highly simplified, driven mainly by a few core technical metrics (such as scoring and shooting percentage). Prior research notes that athletes at this stage rely more on accumulated technical consistency and experience, while age-related physical decline and injury risk make strategic intelligence increasingly important for career longevity (8, 31–33). As a result, both performance potential and technical structure show convergence and platform effects. In practice, when response functions plateau at higher levels or show slight negative slopes (e.g., marginal effects near zero beyond >10–12 points or >3–4 assists per game), training and management should prioritize maintenance: center on stabilizing FG_PCT and defensive positioning, pair with individualized recovery and load management, and sustain on-court contribution at lower physiological cost.

The strong performance of KAN in this study supports theoretical claims that KAN excels at identifying key variables and capturing dynamic nonlinear interactions in complex systems. Methodologically, the study validates the applicability of KAN in sports science and contributes to a quantitative understanding of Long-Term Athlete Development (LTAD), especially in characterizing nonlinear evolution across age stages. These findings also extend and enrich existing frameworks on the Relative Age Effect (RAE) and LTAD (2, 34). Crucially, we link KAN attribution weights with response-function shapes to create a training and deployment decision path: first, use attribution to identify each player's top three primary features (e.g., PTS, AST, REB, FG_PCT) and locate the current level on the corresponding response curves; next, judge the local region by slope, inflection, and plateau—allocate more training/usage in rising segments, organize short threshold-oriented cycles near inflection regions, and shift to efficiency maintenance and load management on plateaus; after each training cycle, reassess on the same curves.

Despite the theoretical and methodological contributions of this study, several limitations remain. First, the inclusion criterion that excluded player–seasons with fewer than 10 min or 10 points per game may introduce selection bias toward higher-performing players, thereby inflating apparent effect sizes and weakening model calibration in low-usage contexts. Second, the model has not incorporated training load, injury status, or psychological and physiological indicators, which may limit predictive accuracy and explanatory depth. The mechanisms are twofold: first, load and health status often co-vary with box-score statistics such as PTS and FG_PCT, making it easy to misattribute state factors as technical factors; second, different levels of fatigue or psychological state can alter the marginal relationships between these statistics and performance, leading to unstable predictions when states change (35, 36). Therefore, future research can proceed along several lines: on the data side, introduce multimodal information—such as sports-physiology and psychological indicators—to increase information content and enhance applicability across contexts; on top of the current KAN pipeline, combine structural equation modeling (SEM) to verify the pathway from training load to technical execution to game performance (37); and, in experimental management, follow engineering best practices, including version-controlled code, preregistered stopping rules, and fold-locked preprocessing within each cross-validation fold (38). In terms of modeling goals, one may draw on ANN-based trajectory modeling from men's 100-m sprinting to explore multi-objective formulations that jointly capture offensive and defensive outputs (39); and incorporate validated, AI-enabled organizational management scales as covariates or moderators in age-group-specific models (40, 41).

Moreover, this study is based solely on NBA data and has yet to evaluate the generalizability of the proposed method across other professional leagues or developmental/amateur systems. Future work should extend this approach to diverse temporal, cultural, and competitive contexts to assess whether the three-phase structure and KAN-derived nonlinear patterns exhibit stable and transferable properties across broader settings.

## 5 Conclusion

This study employed Kolmogorov–Arnold Networks (KAN) to conduct systematic nonlinear modeling and attribution of NBA players' seasonal performance across age groups. The results reveal distinct age-related phases and their key drivers: Youth (19–23 years) show strong potential for nonlinear gains driven by the synergy among multiple technical indicators; Prime (24–30 years) exhibit near-linear, stable responses and a coordinated skill system (a “technical stability plateau”); Veteran (31–40 years) rely on a small set of core skills, with overall performance bounded by a clear ceiling effect. These findings highlight the age-group-specific roles and evolving impact of technical indicators across an athlete's career.

Beyond predictive gains and interpretability, we translate KAN outputs into directly executable guidance: use attribution weights to identify each player's primary features, read the local shape of the corresponding response functions (slope, inflection, plateau), and choose among three actions—invest in the feature when the segment is rising, run short threshold-oriented cycles near inflection regions, or shift to efficiency maintenance and load management on plateaus—then re-evaluate on the same curves after each micro-cycle. As the target metric is Fantasy Points, these age-group-specific patterns should be interpreted as relative effects under this weighting lens, rather than a complete characterization of overall player value.

Future work may incorporate longitudinal tracking, injury histories, team dynamics, and other contextual factors to extend the model's applicability across leagues, genders, and cultural contexts—ultimately strengthening the theoretical and practical foundations for lifecycle-based athlete management and evidence-driven talent selection.

## Data Availability

The original contributions presented in the study are included in the article/Supplementary Material, further inquiries can be directed to the corresponding author.
