# Supplementary material for: Nonlinear age effects on basketball player performance: insights from Kolmogorov–Arnold Networks in NBA data
Source: Front Sports Act Living. 2025 Nov 3;7:1693433. doi: 10.3389/fspor.2025.1693433 (PMC12620410; doi:10.3389/fspor.2025.1693433)
Supplement: Supplementary file 1 [file Table1.docx]

Supplementary Material

# Supplementary Figures and Tables

**Table 1.** All Variables Related to Game Performance and Their Definitions.

| **Variable** | **Definition** |
| --- | --- |
| GP | Number of games played by the player in the season |
| W | Number of games won by the player's team when the player was on the court |
| L | Number of games lost by the player's team when the player was on the court |
| W_PCT | Win percentage in games where the player appeared |
| MIN | Average minutes played per game |
| FGM | Field goals made (including both 2-point and 3-point shots) |
| FGA | Field goals attempted |
| FG_PCT | Field goal percentage |
| FG3M | Three-point field goals made |
| FG3A | Three-point field goals attempted |
| FG3_PCT | Three-point shooting percentage |
| FTM | Free throws made |
| FTA | Free throws attempted |
| FT_PCT | Free throw percentage |
| OREB | Total offensive rebounds |
| DREB | Total defensive rebounds |
| REB | Total rebounds |
| AST | Assists (passes leading directly to a teammate's score) |
| TOV | Turnovers (loss of ball possession due to errors) |
| STL | Steals (gaining possession from the opponent) |
| BLK | Blocks (defensive plays deflecting opponent's shot attempts) |
| BLKA | Times the player's shot was blocked |
| PF | Personal fouls committed |
| PFD | Personal fouls drawn (fouls committed by opponents against the player) |
| PTS | Total points scored during the season |
| PLUS_MINUS | Team point differential while the player is on the court |
| NBA_FANTASY_PTS | Composite performance score calculated using standard Fantasy scoring weights |


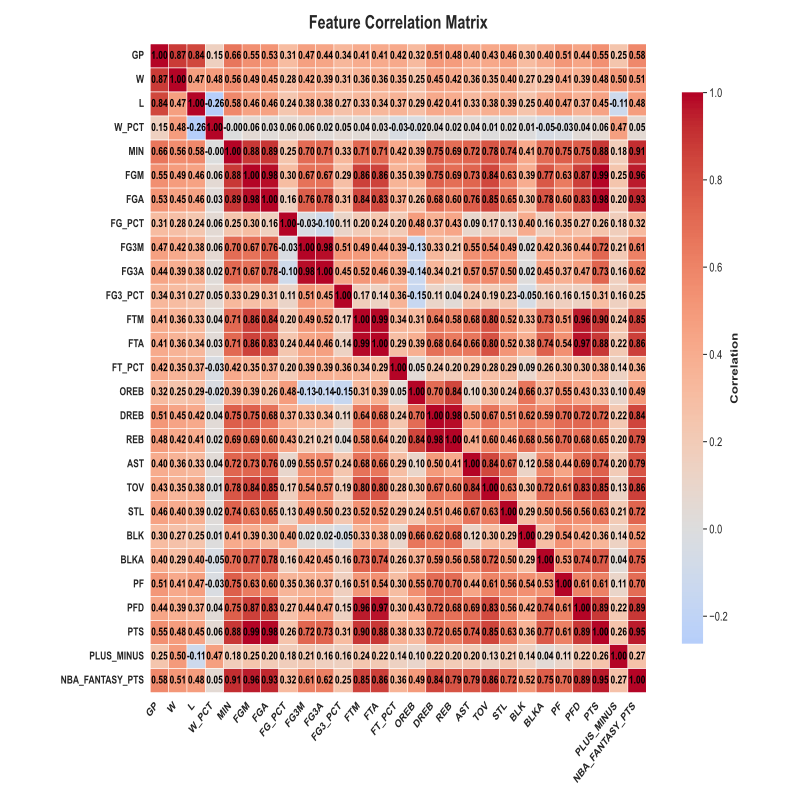
 **Figure 1.** Heatmap of NBA Game Technical Statistics.

**Table 2.** Key Modeling Variables and Their Statistical Characteristics.

| **Variable** | **Mean** | **Std** | **Min** | **25%** | **50%** | **75%** | **Max** |
| --- | --- | --- | --- | --- | --- | --- | --- |
| GP | 59.64 | 16.89 | 1 | 54 | 63 | 71 | 84 |
| MIN | 29.85 | 4.39 | 16.6 | 27 | 30.2 | 33.4 | 43.5 |
| PTS | 13.43 | 4.32 | 6 | 10 | 12.6 | 16.275 | 29.6 |
| FGA | 15.69 | 5.39 | 6.6 | 11.4 | 14.4 | 19 | 34.7 |
| FG_PCT | 0.47 | 0.06 | 0.271 | 0.434 | 0.461 | 0.497 | 0.736 |
| FG3M | 1.76 | 0.93 | 0 | 1.2 | 1.8 | 2.4 | 5.3 |
| FG3_PCT | 0.35 | 0.08 | 0 | 0.329 | 0.364 | 0.39 | 1 |
| FT_PCT | 0.80 | 0.09 | 0 | 0.75 | 0.811 | 0.855 | 1 |
| OREB | 1.11 | 0.84 | 0.1 | 0.5 | 0.8 | 1.4 | 4.7 |
| DREB | 4.22 | 1.87 | 1 | 2.9 | 3.7 | 5.1 | 11.4 |
| REB | 5.33 | 2.54 | 1.2 | 3.5 | 4.6 | 6.575 | 15.2 |
| AST | 3.57 | 2.12 | 0.3 | 1.8 | 3 | 5 | 11.7 |
| TOV | 1.87 | 0.83 | 0 | 1.2 | 1.7 | 2.4 | 4.8 |
| STL | 0.91 | 0.35 | 0 | 0.7 | 0.9 | 1.1 | 2.2 |
| BLK | 0.56 | 0.48 | 0 | 0.3 | 0.4 | 0.7 | 3.6 |
| NBA_FANTASY_PTS | 30.69 | 9.01 | 13.3 | 23.8 | 29.15 | 36.875 | 61.5 |


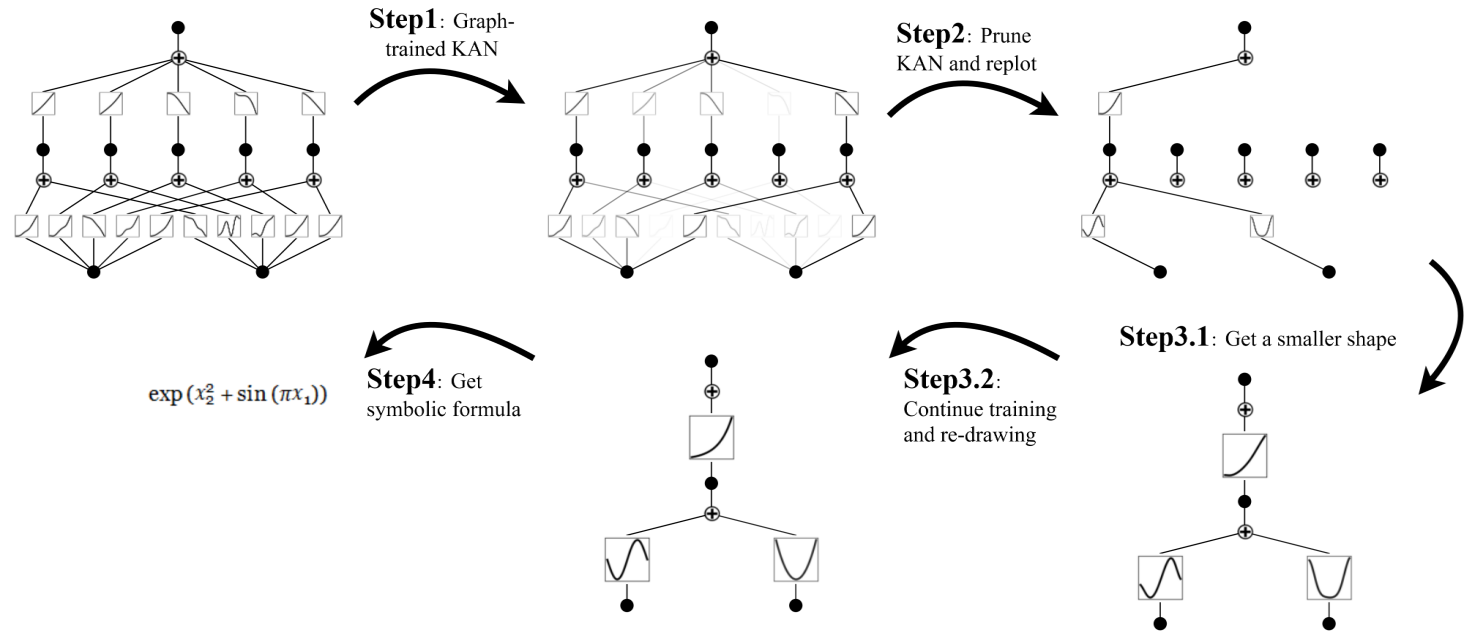


**Figure 2.** Schematic Diagram of the KAN Network Architecture and Symbolic Modeling Workflow.

**Table 3.** Comparison of Predictive Performance Across Models for Players Aged 19–23.

| **Model** | **MAE（Mean ± Standard Deviation）** | **RMSE（Mean ± Standard Deviation）** | **R2（Mean ± Standard Deviation）** |
| --- | --- | --- | --- |
| KAN | **0.0890 ± 0.0117** | **0.1152 ± 0.0159** | 0.9855 ± 0.0038 |
| Linear Regression | 0.6885 ± 0.0140 | 0.9024 ± 0.0230 | **0.9856 ± 0.0038** |
| Random Forest | 1.6755 ± 0.2735 | 2.2805 ± 0.3003 | 0.9107 ± 0.0234 |
| XGBoost | 1.1343 ± 0.0473 | 1.5871 ± 0.2377 | 0.9528 ± 0.0219 |
| MLP | 0.8097 ± 0.1636 | 1.1297 ± 0.2965 | 0.9777 ± 0.0109 |

^Note: Bolded values indicate the best result in each column.^

**Table 4.** Comparison of Predictive Performance Across Models for Players Aged 23–30.

| **Model** | **MAE（Mean ± Standard Deviation）** | **RMSE（Mean ± Standard Deviation）** | **R2（Mean ± Standard Deviation）** |
| --- | --- | --- | --- |
| KAN | **0.0299 ± 0.0030** | **0.0396 ± 0.0033** | **0.9984 ± 0.0002** |
| Linear Regression | 0.7612 ± 0.0736 | 1.0278 ± 0.1127 | 0.9873 ± 0.0018 |
| Random Forest | 1.5177 ± 0.1788 | 2.0998 ± 0.2879 | 0.9459 ± 0.0154 |
| XGBoost | 0.8189 ± 0.0457 | 1.1300 ± 0.0909 | 0.9846 ± 0.0021 |
| MLP | 2.5292 ± 2.2419 | 3.3061 ± 2.5716 | 0.7791 ± 0.2569 |

^Note: Bolded values indicate the best result in each column.^

**Table 5.** Comparison of Predictive Performance Across Models for Players Aged 30–40.

| **Model** | **MAE（Mean ± Standard Deviation）** | **RMSE（Mean ± Standard Deviation）** | **R2（Mean ± Standard Deviation）** |
| --- | --- | --- | --- |
| KAN | **0.0663 ± 0.0563** | **0.0841 ± 0.0713** | **0.9895 ± 0.0159** |
| Linear Regression | 0.9658 ± 0.1027 | 1.2668 ± 0.1496 | 0.9819 ± 0.0034 |
| Random Forest | 1.7332 ± 0.2100 | 2.3258 ± 0.4409 | 0.9389 ± 0.0177 |
| XGBoost | 1.2259 ± 0.1410 | 1.7223 ± 0.3637 | 0.9665 ± 0.0109 |
| MLP | 1.1162 ± 0.2002 | 1.7047 ± 0.5555 | 0.9658 ± 0.0195 |

^Note: Bolded values indicate the best result in each column.^


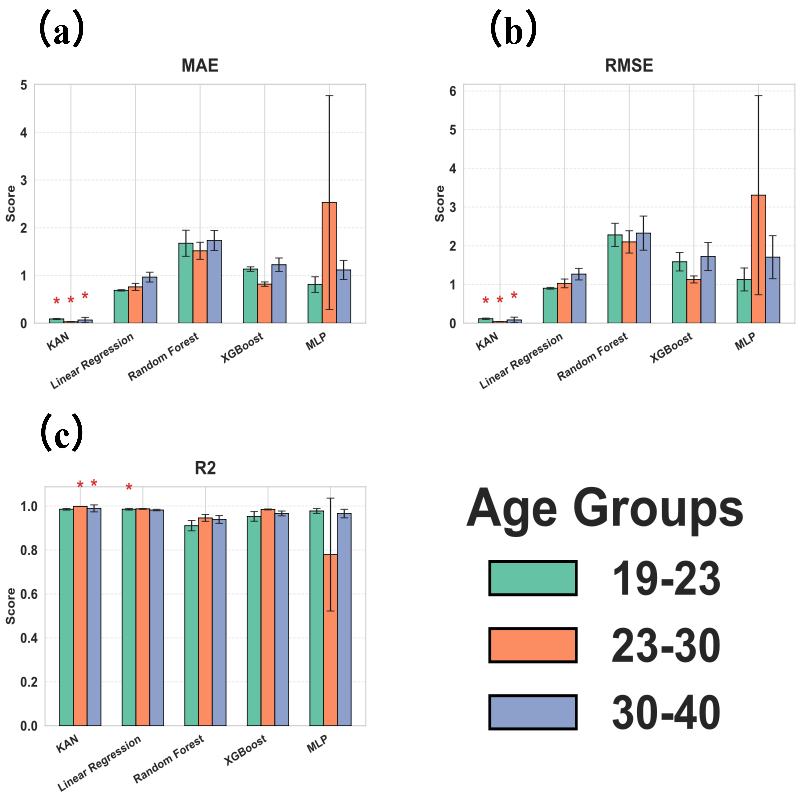


^Note: Asterisks indicate the best result in each column.^

**Figure 3.** Comparison of Predictive Performance Across Age Groups for Different Models (MAE, RMSE, R²).


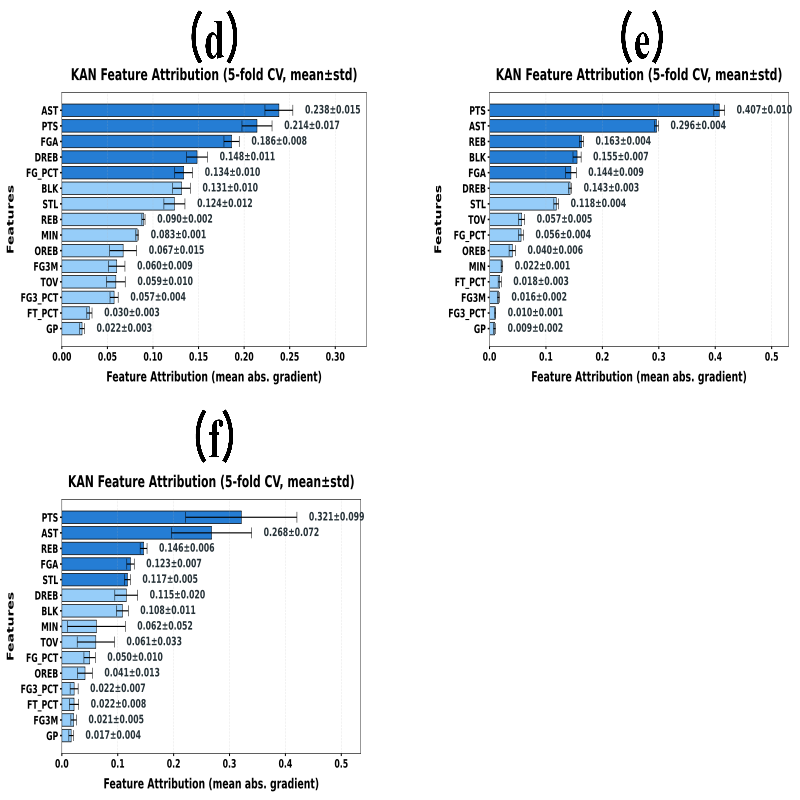


(a) Attribution for 19-23 group; (b) Attribution for 23-30 group; (c) Attribution for 30-40 group. Error bars denote standard deviation.

**Figure 4.** Feature Attribution Distributions from the KAN Model Across Player Age Groups.


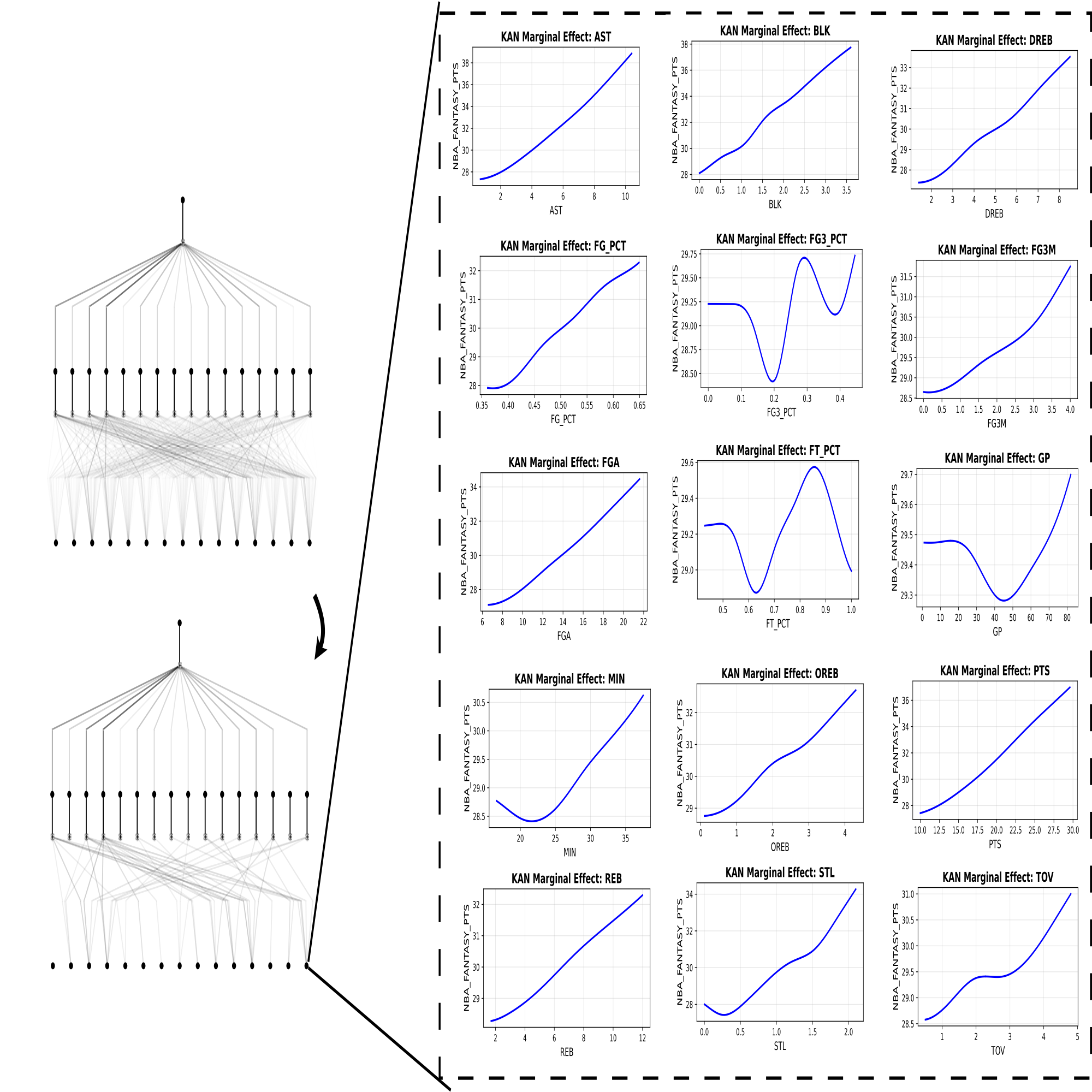


**Figure 5.** Pruned KAN Structure and Dominant Feature Response Functions for Players Aged 19–23.


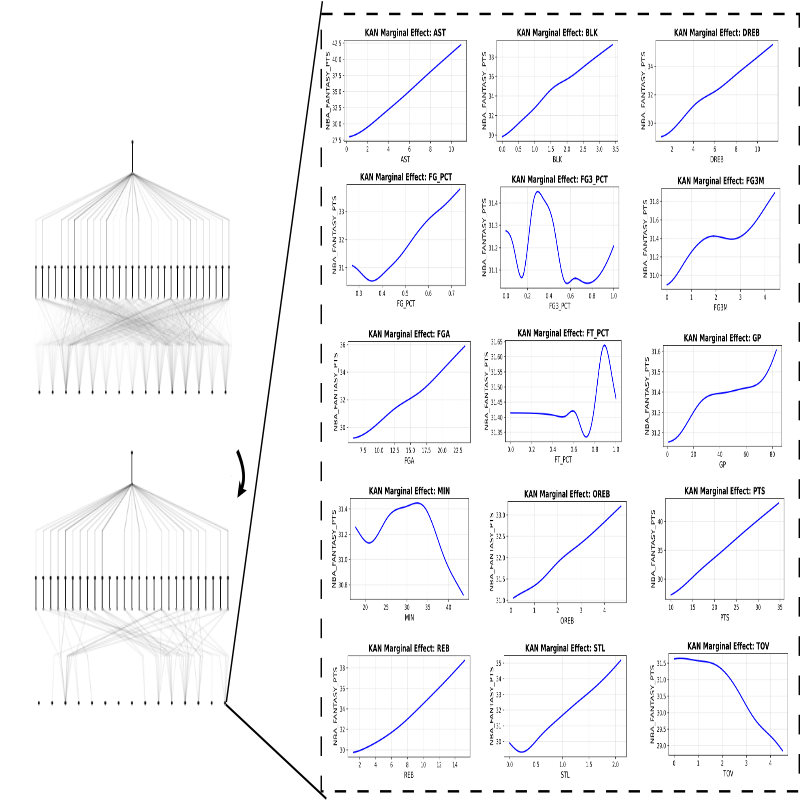


**Figure 6.** Pruned KAN Structure and Dominant Feature Response Functions for Players Aged 24–30.


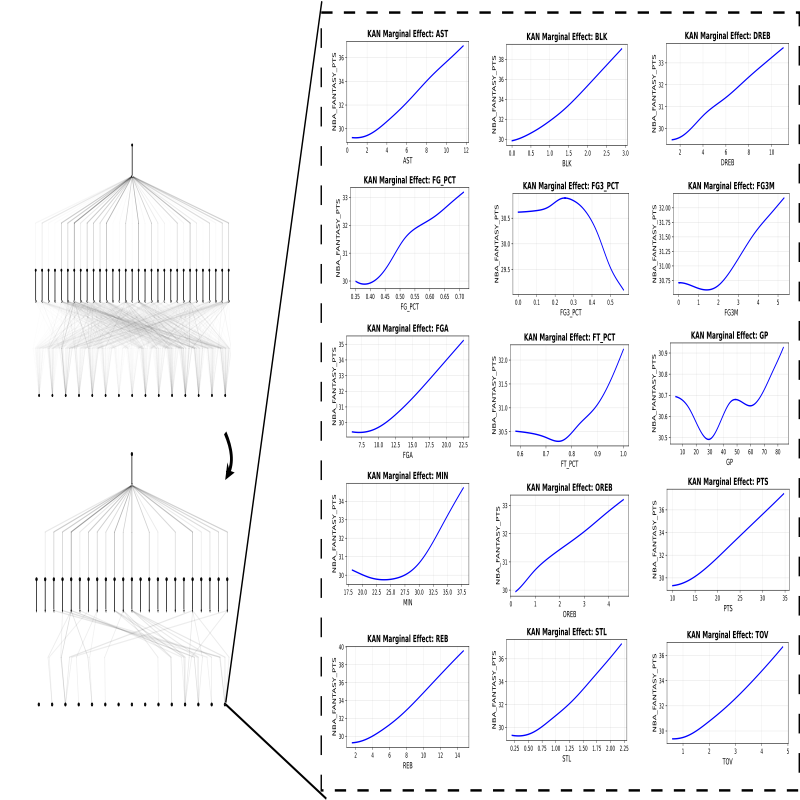


**Figure 7.** Pruned KAN Structure and Dominant Feature Response Functions for Players Aged 31–40.
